# Enhanced Adhesion of Fish Ovarian Germline Stem Cells on Solid Surfaces by Mussel-Inspired Polymer Coating

**DOI:** 10.3390/md17010011

**Published:** 2018-12-26

**Authors:** Yeonwoo Jeong, Jun Hyung Ryu, Yoon Kwon Nam, Seung Pyo Gong, Sung Min Kang

**Affiliations:** 1 Department of Chemistry, Chungbuk National University, Chungbuk 28644, Korea; ywjeong9104@gmail.com; 2 Department of Fisheries Biology, Pukyong National University, Busan 48513, Korea; lion5299@naver.com; 3 Department of Marine Biomaterials and Aquaculture, Pukyong National University, Busan 48513, Korea; yoonknam@pknu.ac.kr

**Keywords:** mussel, polymer coating, polydopamine, ovarian germline stem cell, enhanced adhesion, fish

## Abstract

Development of advanced cell culture methods has gained increasing attention because it allows for efficient genetic engineering and precise regulation of animal reproduction on a cellular basis. Numerous studies have attempted to develop an advanced cell culture method. Previous studies have altered cell culture media and pretreated culture plates with functional molecules. Among them, a mussel-inspired polymer coating has been extensively utilized owing to its wide applicability. For instance, adhesion of human embryonic stem cells and neuronal cells on solid surfaces has been improved. Despite the excellent capability of the mussel-inspired polymer coating, most studies have primarily focused on mammalian cells. However, the efficacy of these coatings on the adhesion of other cell lines is yet unclear. This study aimed to assess the potential of the mussel-inspired polymer coating in the regulation of the adhesion of fish ovarian germline stem cells on solid surfaces. Solid surfaces were coated by polydopamine and poly-L-lysine, and the effect of the coatings on cellular behaviors was investigated.

## 1. Introduction

Traditional seed production for fish has several drawbacks, e.g., it requires a large rearing space and long generation times [1]. In vitro culture of germline stem cells followed by transplantation into recipient fish has been developed to address the issue [2,3,4,5,6,7,8,9,10,11]. For the successful application of this approach, development of advanced in vitro culture methods facilitating the stable expansion of cells and their long-term maintenance are crucial. Previous studies in this field have primarily focused on replacement/optimization of cell culture media [1,12,13,14]. For example, Yoshizaki et al. reported that the addition of bovine serum albumin and adenosine in the culture medium enhanced the viability and transplantability of spermatogonia [1]. As a different approach, surface modification/functionalization of cell culture dishes would significantly improve in vitro culturing of germline stem cells because the chemical composition of solid surfaces significantly affects cell adhesion [15,16]. Surface modifications enabling intimate interactions between cells and cell culture dishes support cell adhesion. In the case of other mammalian cell lines, this surface modification-based approach has already been studied in the regulation of cell adhesion, proliferation, and differentiation [17,18,19,20,21,22,23,24,25,26,27,28]. Specifically, mussel-inspired surface modifications, wherein solid surfaces are treated with adhesive catecholamine derivatives, have been extensively utilized during cell culturing, owing to its wide applicability [29]. The method was inspired by the pivotal role of lysine and 3,4-dihydroxy-L-phenylalanine in the wet adhesion of marine mussels. Catecholamines such as dopamine and norepinephrine have been considered as mussel-inspired molecules and utilized for multifunctional surface coatings [29,30,31,32,33]. For instance, Lee et al. developed a feeder-free system to culture human embryonic stem cells [26]. They synthesized catechol-conjugated heparin and used it for surface coating of cell culture dishes; consequently, cell adhesion was enhanced on the coated surface. Furthermore, Choi et al. developed a mussel-inspired method for improving neuronal adhesion in vitro [19]. Enhanced neuronal adhesion was observed on solid surfaces after coating the surfaces with mussel-inspired polydopamine and poly-L-lysine (PLL). Despite the excellent capability of mussel-inspired surface coatings, most studies have primarily focused on mammalian cells. The efficacy of the coatings on the adhesion of other cell lines including fish germline stem cells is yet unclear.

This study aimed to explore the potential of mussel-inspired polymer coating in enhancing the adhesion of fish ovarian germline stem cells (OGSCs). Solid surfaces were coated with polydopamine and PLL. After characterizing the coated surfaces via X-ray photoelectron spectroscopy and water contact angle goniometry, adhesion assays were conducted on surfaces, using OGSCs derived from Japanese medaka (*Oryzias latipes*).

## 2. Experimental Section

### 2.1. Materials

Dopamine hydrochloride (98%, Sigma Aldrich, St. Louis, MO, USA), Trizma base (99%, Sigma, St. Louis, MO, USA), Trizma·HCl (99%, Sigma, St. Louis, MO, USA), Poly-L-lysine hydrobromide (mol wt. 15,000~30,000 by viscosity, Sigma, St. Louis, MO, USA), and ethanol (94% Samchun, Pyeongtaek, Korea) were used as received.

### 2.2. Animals

Japanese medaka (*Oryzias latipes*) were maintained in the Laboratory of Cell Biotechnology, Pukyong National University (Busan, Korea). Fish were reared in freshwater at 26 °C under a 14:10 h light-dark cycle. All experiments using fish were conducted in compliance of ethical guidelines published by the International Council for Laboratory Animal Science (ICLAS) and the Institutional Animal Care and Use Committee (IACUC) of Pukyong National University approved our research proposal (approval number: 2016-07).

### 2.3. Polydopamine (pDA) Coating

Polystyrene (PS) cell culture dishes were cleaned with ethanol via sonication for 5 min. Cell culture dishes were then treated with a plasma cleaner (Plasma cleaner model PDC-002HARRICK PLASMA, Ithaca, NY, USA) for 5 min. Dopamine was dissolved in tris buffer (pH 8.5, 2 mg/mL) and the solution was transferred to cell culture dishes. The cell culture dishes containing dopamine solution were incubated with constant agitation at room temperature for 1 hour. pDA-coated dishes were rinsed with deionized water and dried under a stream of nitrogen gas.

### 2.4. Immobilization of PLL

A buffered solution of PLL (0.1 mg/mL, tris buffer pH 8.5 10 mM) was added to the pDA-coated dishes, and the dishes were incubated with constant agitation at room temperature for 1 hour. The PLL-immobilized surfaces were rinsed with deionized water and dried under a stream of nitrogen gas. The PLL-immobilized dishes (i.e., pDA/PLL-coated dishes) were refrigerated prior to use.

### 2.5. Enrichment of Ovarian Germline Stem Cells (OGSCs)

To enrich OGSCs, ovaries from 10 adult female *O. latipes* were disintegrated via treatment with 500 U/mL collagenase I (Worthington Biochemical Corporation, Lakewood Township, NJ, USA) dissolved in Leibovitz’s L-15 (L15; Gibco, Grand Island, NY, USA) for 1 hour at 28 °C. The dissociated cells were filtered through a 40-μm cell strainer (Falcon, Corning, NY, USA) to eliminate cellular debris and harvested via centrifugation (400 × g, 4 min). The cells were re-suspended in 0.3 mL Dulbecco’s phosphate-buffered saline (DPBS; Gibco, Grand Island, NY, USA) and subjected to Percoll (Sigma-Aldrich, St. Louis, MO, USA) density gradient centrifugation in accordance with the manufacturer’s instructions. The cells were placed atop a discontinuous 6-step Percoll gradient including 1 mL each of 20%, 25%, 30%, 35%, 40%, and 50% in DPBS and centrifuged at 800× *g* for 30 min. Thereafter, 20% to 40% density fractions containing abundant OGSCs were retrieved and subsequently subjected to differential plating. The cells were washed twice with DPBS and re-suspended in L15 supplemented with 10% (*v*/*v*) fetal bovine serum (FBS; Gibco, Grand Island, NY, USA) and 1% (*v*/*v*) penicillin and streptomycin (P/S; Gibco, Grand Island, NY, USA). Thereafter, 2 to 5 × 10^6^ live cells were seeded in 35-mm dishes (SPL Life Sciences, Pocheon, Korea) coated with 0.1% (*w*/*v*) gelatin (Sigma-Aldrich, St. Louis, MO, USA) and incubated for 15 h at 28 °C with an air atmosphere. Thereafter, floating or loosely-adhered cells were harvested and used for further experiments.

### 2.6. Cell Culture

Enriched OGSCs were equally distributed on non-treated (bare), pDA-coated, and pDA/PLL-coated dishes, and cultured at 28 °C with an air atmosphere. Prior to OGSC culture, the dishes were sterilized via UV irradiation for at least 30 min and pre-incubated with culture medium for 24 h at 28 °C. The composition of culture media was as follows: L15 supplemented with 25 mM HEPES (Sigma-Aldrich, St. Louis, MO, USA), 1% (*v*/*v*) P/S, 6 mg/mL D-(+)-glucose (Sigma-Aldrich, St. Louis, MO, USA), 1% (*v*/*v*) glutamax (Gibco, Grand Island, NY, USA), 1% (*v*/*v*) non-essential amino acids (Gibco, Grand Island, NY, USA), 0.5% (*w*/*v*) bovine serum albumin (Sigma-Aldrich, St. Louis, MO, USA), 5% (*v*/*v*) FBS, 0.25% (*v*/*v*) trout serum (Caisson Laboratories, Smithfield, UT, USA), 1 μg/mL medaka embryo extract, 25 μg/mL bovine insulin (Sigma-Aldrich, St. Louis, MO, USA), 100 μg/mL recombinant human apo-transferrin (Sigma-Aldrich, St. Louis, MO, USA), 10 ng/mL recombinant human basic fibroblast growth factor (Gibco, Grand Island, NY, USA), 10 ng/mL recombinant human glial cell-derived neurotrophic factor (Peprotech, Rocky Hill, NJ, USA), 5% (*v*/*v*) Knockout serum replacement (Gibco, Grand Island, NY, USA), 50 μM ascorbic acid (Sigma-Aldrich, St. Louis, MO, USA), 50 μM β-mercaptoethanol (Gibco, Grand Island, NY, USA), and 2 nM sodium selenite (Sigma-Aldrich, St. Louis, MO, USA). Half of the culture media were replenished every 3 days. The status of cultured cells was checked daily with an inverted microscope (TS-100F, Nikon, Tokyo, Japan) and cell adherence was confirmed by evaluating whether the cultured cells were floating and moving or not when the culture plate was manually agitated. Embryonic cell line (somatic cells) was established from *O. latipes* embryos at 32 to 36 stages according to conventional method and cultured in L15 supplemented with 20% (*v*/*v*) FBS and 1% (*v*/*v*) P/S on 0.1% (*w*/*v*) gelatin-coated culture plates at 28 °C in an air atmosphere. The embryonic cells at passage 30 were used for analysis.

### 2.7. Cell Viability

To evaluate cell viability after culturing the enriched OGSCs under different substrate conditions, cells were seeded in 4-well plates (SPL Life Sciences, Pocheon, Korea) at a density of 1.5 × 10^4^ live cells per well and cultured for 10 days. The cultured cells were harvested via treatment of 0.05% trypsin-EDTA (Gibco, Grand Island, NY, USA) and the live cells were enumerated after staining with Trypan Blue (Gibco, Grand Island, NY, USA). The survival rate (%) was calculated as follows: the number of live cells after culture / the number of live cells initially seeded × 100.

### 2.8. Cell Transplantation

For cell transplantation, 5 × 10^5^ live cells after OGSC enrichment were labeled with red fluorescence, using 6 µM PKH26 (Sigma-Aldrich, St. Louis, MO, USA) and cultured on different substrate conditions for 10 days. After cell retrieval, 3 × 10^3^ live cells were transplanted into the peritoneal cavity of recipient *O. latipes* larvae 11 days post fertilization (dpf). After 9 days, colonies of transplanted cells in the gonadal region of developing recipient larvae were observed using a TS-100F microscope equipped with a fluorescence unit.

### 2.9. Reverse Transcription Polymerase Chain Reaction (RT-PCR) and Quantitative RT-PCR (qRT-PCR)

Total RNA from the enriched OGSCs cultured for 7 days was extracted using the RNeasy Plus Micro Kit (Qiagen, Valencia, CA, USA). The cDNA was synthesized from 150 ng total RNA using the GoScript reverse transcription system (Promega, Madison, WI, USA) after treatment with DNase I (Sigma-Aldrich, St. Louis, MO, USA) according to the manufacturer’s instructions. Sequence-specific primers for *nanos2*, *vasa*, and *β-actin* were designed using the Primer-BLAST program (https://www.ncbi.nlm.nih.gov/tools/primer-blast/), and their sequences were shown in Table 1. After PCR amplification with specific primers, the PCR products were size-fractionated by 1.2% agarose gel electrophoresis and visualized by GelRed (Biotium, Hayward, CA, USA). Quantitative reverse transcription polymerase chain reaction (qRT-PCR) was performed using a LightCycler 480 II Real-Time PCR System (Roche Applied Science, Mannheim, Germany) with a LightCycler 480 SYBR Green I Master (Roche Applied Science, Mannheim, Germany). *β-actin* mRNA level was used for normalizing the specific gene expression. PCR condition was as follows; 45 cycles of 95 °C for 10 s, 60 °C for 20 s, and 72 °C for 20 s. The mRNA level of each gene was presented as 2^-ΔΔCt^, where Ct = the threshold cycle for target amplification, ΔCt = Ct_target gene_ – Ct_internal reference (*β-actin*)_, and ΔΔCt = ΔCt_sample_ – ΔCt_calibrator_.

### 2.10. Characterizations

X-ray photoelectron spectroscopy (XPS) was carried out using a PHI Quantera II (ULVAC-PHI, Inc., Japan) with an Al Kα X-ray source and under ultrahigh vacuum (~10^−10^ mbar). Static water contact angles were measured using a Phoenix-300 TOUCH goniometer (Surface Electro Optics Co., Ltd., Korea).

### 2.11. Statistical Analysis

Data are presented as mean ± standard deviation values. A t-test and analysis of variance (ANOVA) followed by Duncan method were performed to compare the survival rates and gene expression levels of OGSCs after culture, respectively, using SPSS program (IBM-SPSS, Chicago, IL, USA).

Gonadal colonization efficiencies of the transplanted OGSCs were analyzed in accordance with the treatments, using analysis of variance, followed by least squares analysis, using Statistical Analysis System software (SAS Institute, Cary, NC, USA).

## 3. Results and Discussion 

**Figure 1:**
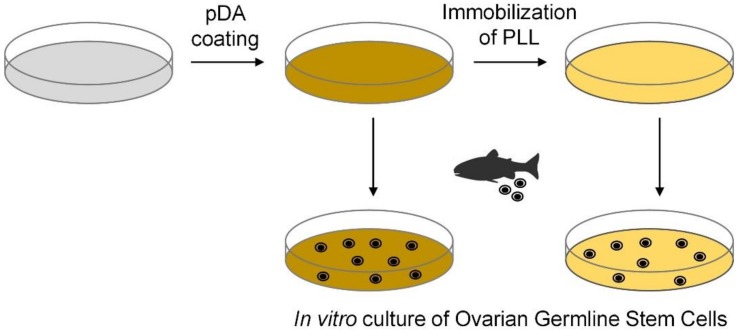
Schematic representation of the polydopamine (pDA) and pDA/ poly-L-lysine (PLL) coatings on PS cell culture dishes.

Cell culture dishes were surface-modified using mussel-inspired technology. In particular, dopamine, a known small-molecule mimic of mussel-adhesive proteins [29] was used to modify the surface characteristics of cell culture dishes. Under alkaline conditions, dopamine is converted to pDA via oxidation and self-polymerization [29]. Because pDA adheres on various surfaces and functional molecules can be grafted to pDA, pDA coating has been extensively utilized during surface functionalization/modification [30,31]. Herein, PS cell culture dishes were modified with pDA and PLL for application in in vitro OGSC culturing (Figure 1).

**Figure 2:**
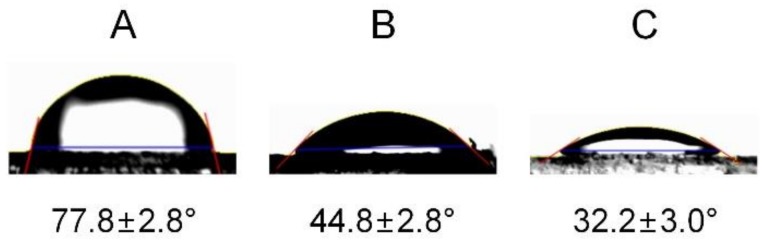
Water contact angle images of (**A**) non-treated, (**B**) pDA-coated, and (**C**) pDA/PLL-coated surfaces.

Surface modification with pDA and PLL was characterized via water contact angle goniometry and XPS. The water contact angle of the surface decreased from 77.8 ± 2.8° to 44.8 ± 2.8° and then to 32.2 ± 3.0° after pDA coating and PLL immobilization, indicating an increase in surface hydrophilicity resulting from the introduction of hydrophilic pDA and PLL on the surface (Figure 2) [19]. As a control, non-treated PS surfaces were treated with PLL for 1 hour, but the water contact angle (61.6 ± 3.3°) of the surface was much higher than that for the pDA/PLL-coated surface. It means that the pDA coating is required for efficient immobilization of PLL onto the PS surfaces.

**Figure 3:**
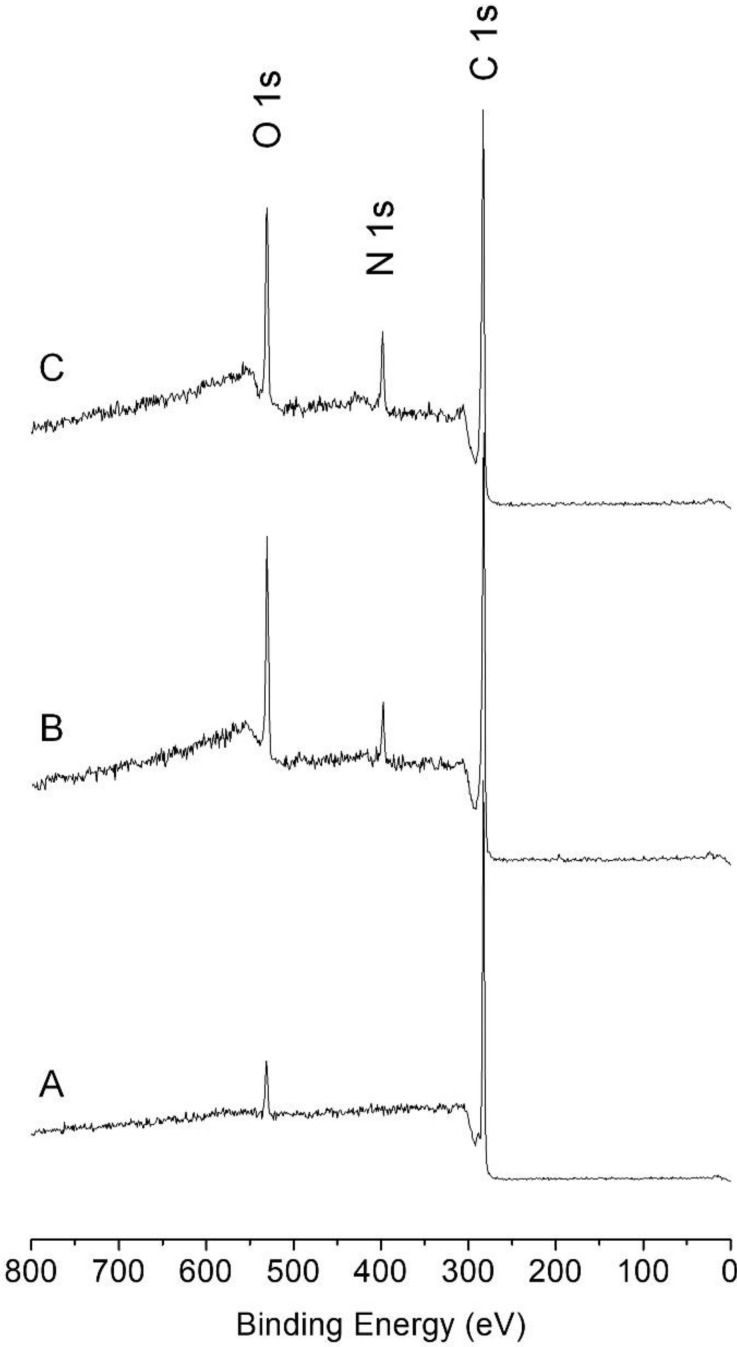
X-ray photoelectron spectra of (**A**) non-treated, (**B**) pDA-coated, (**C**) pDA/PLL-coated surfaces.

Successful modification of PS cell culture dishes with pDA and PLL was also evident from XPS analysis. Unlike the XPS spectrum of a non-treated surface (Figure 3A), the XPS spectrum of the pDA-coated surface showed C 1s, N 1s, and O 1s peaks. (Figure 3B) After immobilization of PLL on the pDA-coated surface, the N 1s peak increased with a concurrent reduction in the peak intensities of O 1s and C 1s (Figure 3C and Table 2). Analysis of the areal ratio between N 1s and O 1s in the pDA-coated surface was 0.32. The ratio increased to 0.54 after the immobilization with PLL, indicating that the organic layer with relatively rich in N, i.e., PLL, was introduced on the pDA-coated surface [19].

**Figure 4:**
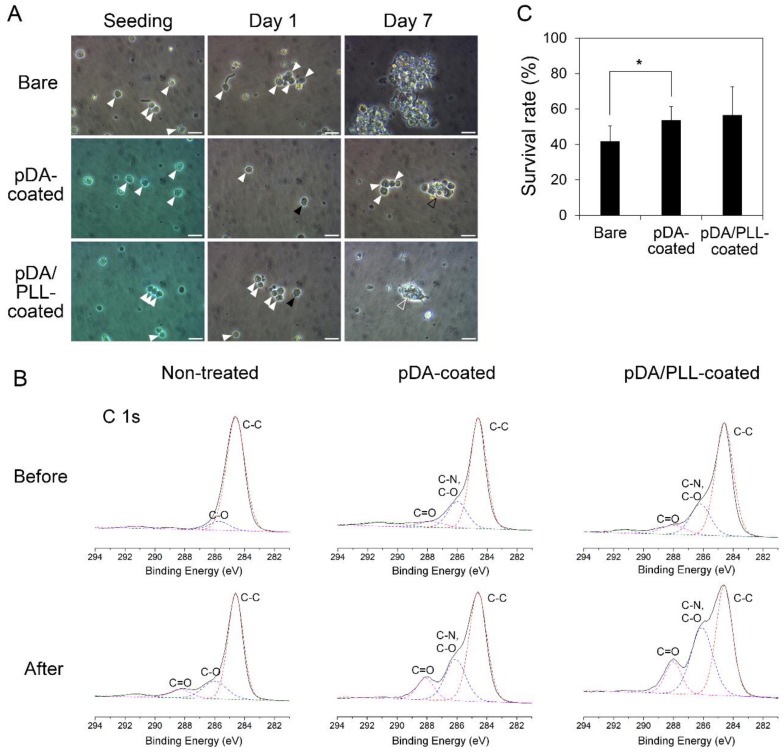
Effects of pDA or pDA/PLL coatings on adherence and survival of the enriched *Oryzias latipes* ovarian germline stem cells (OGSCs) in culture. (**A**) OGSC adherence in culture depending on different substrate conditions. After seeding, numerous OGSCs were observed in all groups (white arrowheads) and after 1 day, a few OGSCs adhered on pDA- and pDA/PLL-coated dishes (black arrowheads), while none of OGSCs adhered on non-treated dishes. On day 7, loosely (black hollow arrowhead) and tightly packed (white hollow arrowhead) OGSC colonies were observed in pDA- and pDA/PLL-coated dishes, respectively, whereas only the floating cells and cell aggregates were observed in non-treated dishes. Scale bar = 20 µm. (**B**) High resolution X-ray photoelectron spectra (C 1s) of non-treated, pDA-coated, and pDA/PLL-coated surfaces before and after incubation in cell culture media. (**C**) Survival rates of the cell populations containing the enriched OGSCs in culture depended on different substrate conditions. The cell survival rate increased significantly when the cells were cultured on pDA-coated dishes rather than when cultured on non-treated dishes. An asterisk (*) indicates a significant difference, *p* < 0.05.

After confirming pDA and PLL coatings, the enriched OGSCs were cultured on non-treated, pDA-coated, and pDA/PLL-coated dishes for 7 days, and the adhesion characteristics were analyzed. As shown in Figure 4A, numerous OGSCs with a large nucleus with one or two prominent nucleoli were observed in all culture dishes after seeding. After 1 day, adherence of few OGSCs was observed in the groups cultured on pDA- and pDA/PLL-coated dishes; however, no OGSC adherence was detected in the group cultured on non-treated dishes. At day 7 of culturing, adherent OGSCs in pDA- and pDA/PLL-coated dishes formed loosely and tightly packed colonies, respectively. On the contrary, the OGSCs cultured on non-treated dishes still did not adhere to the substratum, and some of them formed floating cell aggregates.

These results indicate that both pDA and PLL coatings provide a favorable environment for the initial adhesion of OGSCs, probably resulting from the protein-friendly property of pDA [29]. In order to confirm the protein-friendly property of pDA, all surfaces were analyzed by XPS after 24 h-incubation in cell culture media. Unlike the non-treated PS surfaces, pDA- and pDA/PLL-coated surfaces showed a significant increase of amide carbonyl peak at 288 eV (Figure 4B) [18]. It implies that pDA and pDA/PLL coatings facilitated protein adhesion on surfaces. Protein adhesion on substrata with low surface energies can lead to protein denaturation, thereby disrupting protein-mediated cell adhesion [18]. Hydrophilic conversion of substrates via pDA coating minimizes protein denaturation and facilitates cell adhesion. Regarding the pDA/PLL-coated surface, enhanced adhesion is also attributable to the electrostatic attraction between PLL and cells. Under physiological conditions, PLL is positively-charged; hence, cells with negatively-charged membranes can easily adhere to the pDA/PLL-coated surface.

The cell survival rate was compared between the cell populations cultured under different substrate conditions after 10 days of culturing. In this case, entire cell populations, not OGSCs in particular, were considered for determining the survival rate because the cultured cell populations contained not only the enriched OGSCs but also a small number of non-OGSC cells. Compared with cells seeded initially, the cell number decreased by approximately 58% in the cells cultured on non-treated dishes. However, the survival rate increased significantly when the cells were cultured on pDA-coated dishes (Figure 4C; 53.8 ± 7.5% vs. 41.9 ± 8.6%, *p* = 0.047). Cells cultured on pDA/PLL-coated dishes also displayed a slightly but not significantly increased the survival rate of 56.6 ± 15.9% in comparison with those cultured on non-treated dishes (*p* = 0.117).

**Figure 5:**
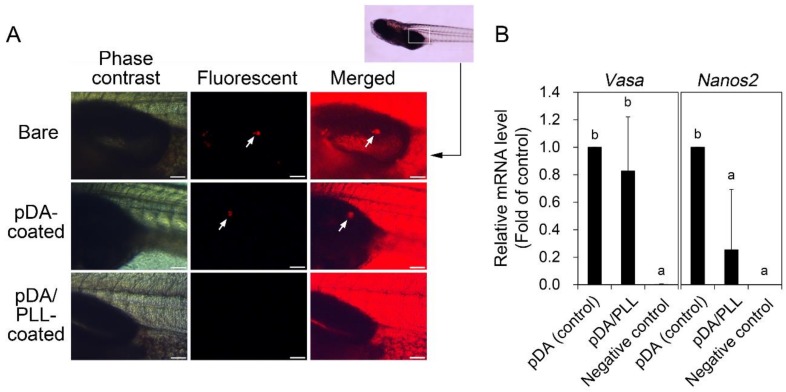
Effects of pDA or pDA/PLL coating on gonadal colonization and relative mRNA expression of *vasa* and *nanos2* genes of ovarian germline stem cells (OGSCs) after culture. (**A**) Gonadal colonization of OGSCs cultured on pDA- and pDA/PLL-coated dishes. The OGSCs cultured for 10 days on pDA- and pDA/PLL-coated dishes were labeled with fluorescent dye (PKH26) and intraperitoneally transplanted into 11 days-post-fertilization (dpf) larvae. After 9 days, gonadal colonization of the transplanted OGSCs was observed in the larvae transplanted with the OGSCs cultured on non-treated and pDA-coated dishes. In contrast, no colonization was observed in larvae transplanted with the OGSCs cultured on pDA/PLL-coated dishes. Arrows indicate the OGSCs colonized. Scale bar = 100 μm. (**B**) Relative mRNA expression of *vasa* and *nanos2* genes between the enriched OGSCs cultured on pDA and pDA/PLL-coated dishes. The enriched OGSCs were cultured on pDA- or pDA/PLL-coated dishes and then the cells were subjected to quantitative reverse transcription polymerase chain reaction (qRT-PCR) analysis. A significant decrease of *nanos2* expression was observed in the cells cultured on pDA/PLL-coated dishes compared to the cells cultured on pDA-coated dishes, whereas no significant difference was detected in *vasa* expression between two cell populations. ^ab^ Different letters indicate significant difference, *p* < 0.05. An *Oryzias latipes* embryonic cell line was used as a negative control.

To determine whether the OGSCs maintain their function to colonize in the developing gonads after culturing, OGSCs cultured on different substrata for 10 days were intraperitoneally transplanted to the developing larvae, and their gonadal colonization was examined. At 20 dpf of the recipient larvae, the survival rate was significantly decreased in the recipient larvae transplanted with the OGSCs cultured on pDA/PLL-coated dishes in comparison with the other treatment groups (Table 3; 50% vs. 71 to 80%, *p* < 0.05). Gonadal colonization of the transplanted OGSCs was observed in two OGSC groups cultured on non-treated and pDA-coated dishes; however, an approximately 2-fold increase in its rate was observed in pDA-coated dishes. In contrast, no gonadal colonization was observed in the larvae transplanted with the OGSCs cultured on pDA/PLL-coated dishes (Figure 5A and Table 3). In this type of transplantation assay, gonadal colonization is a marker for undifferentiated germline cells. Hence, the results imply that pDA-coated dishes play an important role in maintaining the undifferentiated state of OGSCs. However, pDA/PLL-coated dishes might induce the differentiation of OGSCs, thereby decreasing their colonization in the developing gonads. To confirm this, we investigated the expression of *vasa* and *nanos2* genes in the enriched OGSCs cultured on different surface conditions. RT-PCR results showed that both genes expressed in all treatment groups after culture even though their expression signals were weak (Figure S1). These indicate that all conditions used in this study are able to contribute to retaining the stem cell characteristics of OGSCs under the different levels of contribution. Subsequently, we compared the expression level of *vasa* and *nanos2* genes between the enriched OGSCs cultured on pDA- and pDA/PLL-coated dishes by qRT-PCR analysis to verify the difference in the results of cell transplantation assay. As shown in Figure 5B, a significant difference was not detected in *vasa* expression between two cell populations. However, a significant decrease of *nanos2* expression was observed in the cells cultured on pDA/PLL-coated dishes compared to pDA-coated dishes. In *O. latipes* embryonic cells, which were used as a negative control, expression of both genes was hardly detected. As a well-known germ cell marker, *vasa* is expressed in all types of germ cells regardless of differentiation status [34]. In contrast, *nanos2* expression is limited only in the OGSCs [3,35]. Based on this, our qRT-PCR results indicate that proportion of total germ cells was similar between two cell populations after culture, but the cell population cultured on pDA-coated dishes contained a higher proportion of OGSCs than that on pDA/PLL-coated dishes. These imply that pDA/PLL-coated dishes partially contributed to the differentiation of OGSCs while pDA-coated dishes did not do that. Collectively, the present results indicate that it is effective to apply a pDA coating alone for maintaining fish OGSCs in culture.

## 4. Conclusions

In summary, mussel-inspired polymer coatings were used to enhance in vitro culturing of fish OGSCs on solid surfaces. Culture dishes were coated with either pDA or pDA/PLL, and cellular behavior of the OGSCs derived from Japanese medaka was investigated. Compared with cells cultured on non-treated dishes, OGSCs cultured on pDA-coated or pDA/PLL-coated dishes showed higher viability, and pDA coating was superior to the pDA/PLL coating in terms of maintenance of the undifferentiated state of OGSCs. The present results indicate that a pDA coating plays an important supportive role in vitro culturing of OGSCs. We believe that the OGSC-adhesive property of the pDA coating may be further improved by combining pDA with cell adhesion-triggering peptides and proteins.

## Figures and Tables

**Table 1:** Primer sequences used in this study.

Genes	Primer Sequences (5′ > 3′)	Product Size (bp)	Accession Number
*nanos2*	Forward, GGTGCAAACAACTGTGGATG	262	NM_001160447.1
	Reverse, CTTGCAGAAGCGGCAGTAAT		
*vasa*	Forward, GAGAAGGTTCCGACCACCAG	177	NM_001104676.1
	Reverse, AATGGTGTTGGGCAGGTCAA		
*β−actin*	Forward, CCACCATGTACCCTGGAATC	153	NM_001104808.1
	Reverse, GCTGGAAGGTGGACAGAGAG		

**Table 2:** Atomic composition (%) of non-treated, pDA-coated, and pDA/PLL-coated surfaces.

	C 1s	N 1s	O 1s	N 1s/O 1s
Non-treated	95.8	0	4.2	0
pDA-coated	77.4	5.5	17.1	0.32
pDA/PLL-coated	74.8	8.8	16.4	0.54

**Table 3:** Rates of gonadal colonization after intraperitoneal transplantation of *Oryzias latipes* ovarian germline stem cells (OGSCs) cultured on pDA- or pDA/PLL-coated dishes into 11 days-post-fertilization (dpf) larvae.

Substrate conditions of culture dishes	Number of recipients transplanted	Number (%) ^a^ of recipients survived to 20 dpf	Number (%) ^a^ of recipients harboring transplanted OGSCs in their gonads
Non-treated	28	20 (71) ^bc^	3 (11) ^bc^
pDA-coated	30	24 (80) ^b^	6 (20) ^b^
pDA/PLL-coated	30	15 (50) ^c^	0 (0) ^c^

^a^ Percentage of number of recipients transplanted. ^bc^ Different letters within the same column indicate significant differences, *p* < 0.05.

## Supplementary Materials

The following are available online at http://www.mdpi.com/1660-3397/17/1/11/s1, Figure S1: Expression of *vasa* and *nanos2* genes in the enriched ovarian germline stem cells (OGSCs) cultured on different surface conditions.
