# Supplementary material for: Enhanced Adhesion of Fish Ovarian Germline Stem Cells on Solid Surfaces by Mussel-Inspired Polymer Coating
Source: Mar Drugs. 2018 Dec 26;17(1):11. doi: 10.3390/md17010011 (PMC6369427; doi:10.3390/md17010011)
Supplement: Supplementary file 1 [file marinedrugs-17-00011-s001.pdf]

marine drugs

Supplementary Material

Enhanced Adhesion of Fish Ovarian Germline Stem Cells on Solid Surfaces by Mussel-Inspired Polymer Coating

Yeonwoo Jeong, Jun Hyung Ryu, Yoon Kwon Nam, Seung Pyo Gong*, and Sung Min Kang*

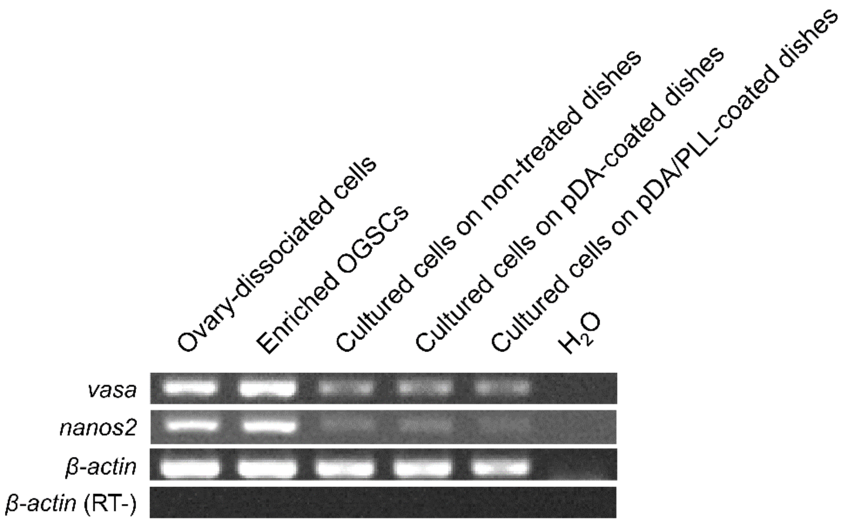

Figure S1. Expression of vasa and nanos2 genes in the enriched ovarian germline stem cells (OGSCs) cultured on different surface conditions. The enriched OGSCs cultured on different surface conditions were collected and were subjected to RT-PCR. Ovary-dissociated cells and enriched OGSCs were used for the positive controls. Weak expression of both genes were detected in all treatment groups after culture.
